# Epidemiological characteristics of first-time SARS-CoV-2 Omicron infection among hospital staff in Chengdu, China

**DOI:** 10.1186/s41043-024-00595-3

**Published:** 2024-07-08

**Authors:** Li Tang, Yeyuan Wang, Xue Li, Liu Yang, Yingjuan Luo, Chunrong Li, Yulei He

**Affiliations:** 1 Chengdu Women’s and Children’s Central Hospital, School of Medicine, University of Electronic Science and Technology of China, Chengdu, 611731 China; 2 School of Medicine, University of Electronic Science and Technology of China, Chengdu, 611731 China

**Keywords:** COVID-19, China, Incidence, Omicron, Associated factors, Symptoms

## Abstract

**Background:**

After China ended its ‘dynamic zero-COVID policy’ on 7 December 2022, a large-scale outbreak of SARS-CoV-2 Omicron infections emerged across the country. We conducted a hospital-wide prospective study to document the epidemiological characteristics of the outbreak among healthcare workers in a hospital of Chengdu, where no previous staff SARS-CoV-2 infections were detected.

**Methods:**

All hospital staff members were invited to complete an online questionnaire on COVID-19 in January 2023, and SARS-CoV-2 infection cases were followed up by telephone in June 2023 to collect data on long COVID. Univariable and multivariable logistic regression analyses were performed to evaluate factors associated with SARS-CoV-2 infection.

**Results:**

A total of 2,899 hospital staff (93.5%) completed the online questionnaire, and 86.4% were infected with SARS-CoV-2 Omicron. The clinical manifestations of these patients were characterized by a high incidence of systemic symptoms. Cough (83.4%), fatigue (79.8%) and fever (74.3%) were the most frequently reported symptoms. Multivariable logistic analysis revealed that females [adjusted odds ratio (aOR): 1.42, 95% confidence interval (CI): 1.07–1.88] and clinical practitioners (aOR: 10.32, 95% CI: 6.57–16.20) were associated with an increased risk of SARS-CoV-2 infection, whereas advanced age ≥ 60 years (aOR: 0.30, 95% CI: 0.19–0.49) and a three-dose COVID-19 vaccination with the most recent dose administered within 3 months before 7 December 2022 (aOR: 0.44, 95% CI: 0.23–0.87 for within 1 month; aOR: 0.46, 95% CI: 0.22–0.97 for within 1–3 months) were associated with reduced risk. Among the cases, 4.27% experienced long COVID of fatigue, brain fog or both, with the majority reporting minor symptoms.

**Conclusion:**

Our findings provide a snapshot of the epidemiological situation of SARS-CoV-2 infection among healthcare workers in Chengdu after China’s deregulation of COVID-19 control. Data in the study can aid in the development and implementation of effective measures to protect healthcare workers and maintain the integrity of healthcare systems during challenging times such as a rapid and widespread Omicron outbreak.

## Introduction

Severe acute respiratory syndrome coronavirus-2 (SARS-CoV-2) and its disease (COVID-19), which first emerged in Wuhan in late 2019, spread rapidly around the world. In response, the World Health Organization (WHO) declared a global pandemic, officially lasted from 11 March 2020 to 5 May 2023. During this time, several SARS-CoV-2 variants were reported globally [1]. The Omicron variant B.1.1.529, initially identified in South Africa in November 2021 [2], and its subvariants quickly became predominant worldwide, leading to an increase in COVID-19 cases [3]. Studies suggest that compared to the delta variants [1], Omicron is more contagious and has greater immune escape capabilities. Its high transmissibility posed challenges for infection control efforts.

On 7 December 2022, China released a 10-point notice to optimize its COVID-19 control policies [4], which brought ‘dynamic zero-COVID policy’ to an end. This sudden policy change resulted in a rapid increase in Omicron SARS-CoV-2 infections throughout China. Chengdu, the capital of Sichuan Province and China’s fourth most populous city, reported its first COVID-19 case on 21 January 2020. At the end of 2022, Chengdu experienced a significant surge in COVID-19 cases and hospitalizations, with the Omicron subvariant BA.5 being the most prevalent strain [5]. The surge in infection cases inevitably placed strain and challenges on healthcare services [6].

Healthcare workers generally face a higher risk of SARS-CoV-2 infection compared to the general population in the community. Previous studies showed significant variability in the incidence of COVID-19 among healthcare workers, primarily due to disparities in viral strains and observation periods. For example, during a two-week recruitment period of a cohort study in the U.S. in 2020, 7.3% of healthcare workers tested positive for SARS-CoV-2 infection [7]. However, following the emergence of the Omicron variant, a notable increase in incidence was observed. Two studies conducted between late 2021 and mid-2022 revealed that the proportion of infected healthcare workers was 24.9% in Hong Kong [8] and 20.3% in Taiwan [9]. Recognizing the factors that expose healthcare workers to a heightened risk of acquiring COVID-19 is crucial. A recent review identified inadequate or lack of protective personal equipment, performing tracheal intubation, and being female as the most common factors associated with COVID-19 infection among healthcare workers [10]. However, some studies found no links between occupational factors and SARS-CoV-2 infection in this population [11, 12]. Regarding long COVID, the incidence also varied widely depending on the methodologies employed [13]. A case-control study of Brazilian healthcare workers showed that 27.4% developed long COVID after infection. Female gender, older age, multiple infections were found to increase the risk, whereas receiving four doses of COVID-19 vaccine prior to infection was a protective factor against long COVID symptoms [14].

Understanding how the virus affects healthcare workers is essential for devising effective strategies to mitigate transmission and protect healthcare workers, thereby ensuring the continuity of essential healthcare services during periods of heightened transmission. However, there are few data on SARS-CoV-2 infection among healthcare workers in mainland China, particularly during a nationwide Omicron outbreak. In the present study, we investigated the cumulative incidence, symptoms and factors associated with SARS-CoV-2 Omicron infection among hospital staff in a tertiary women’s and children’s hospital of Chengdu after China relaxed its COVID-19 restrictions in December 2022. All hospital staff had no evidence of previous SARS-CoV-2 infections. The presence of long COVID symptoms of fatigue and brain fog was also explored in the present study.

## Materials and methods

### Study setting and design

A prospective cohort study of COVID-19 was undertaken among staff working at Chengdu Women’s and Children’s Central Hospital, a university-affiliated tertiary hospital located in Chengdu, Sichuan Province, China. Prior to 7 December 2022, nucleic acid testing was compulsory for every staff member in the hospital at least twice per week, and no SARS-CoV-2 infections were detected.

In the study, eligible participants were those who became full-time staff of Chengdu Women’s and Children’s Central Hospital, either permanent or temporary, before 7 December 2022. Between 17 and 23 January 2023, a message including a link to a self-administered online questionnaire on SARS-CoV-2 infection was sent to all eligible hospital staff via WeCom (previously known as WeChat work), a business communication and office tool. Eligible participants who were willing to participate provided their informed e-consent by ticking an electronic checkbox before filling the questionnaire. In June 2023, the participants with SARS-CoV-2 infection at baseline were followed up by telephone by trained interviewers to collect information on long COVID.

### Questionnaires

The baseline self-administered questionnaire comprises two parts: demographic information and COVID-19-related data. The demographic data of each participant were investigated first, including gender, age, occupation, height, weight and health conditions. Then, detailed information was solicited on the diagnosis, presence of 35 predefined symptoms, severity, complications and vaccination uptake status of COVID-19. The questionnaire used for follow-up focused on two of the commonly reported long COVID symptoms—fatigue and brain fog. The questionnaires were designed based on previously reported research on COVID-19 [15, 16], and the draft was revised by two clinical experts. Before the questionnaires were officially used, small-scale pilot surveys were conducted within the corresponding author’s department. The questionnaires were further improved based on feedback to ensure that all of the questions were clear and understandable.

### Definitions

A confirmed COVID-19 diagnosis was defined as a positive COVID-19 PCR or antigen test on a respiratory sample combined with symptoms suggestive of COVID-19, such as sore throat, cough, and nasal congestion. Probable COVID-19 cases were participants who had a clinical presentation consistent with COVID-19 but had not been tested for SARS-CoV-2. In the present study, a case with SARS-CoV-2 infection was defined as a participant who had a confirmed COVID-19 diagnosis or who was a probable case.

Long COVID was defined as symptoms that started within three months of the initial SARS-CoV-2 infection, lasted for at least two months, and could not be explained by another condition [17]. In our study, brain fog was a range of neurocognitive symptoms, including forgetfulness, sluggish feelings, poor concentration, confusion, fuzzy thoughts, and word loss.

### Data analysis

Data analysis was performed using SPSS 22 (IBM, Armonk, NY, USA). Descriptive statistics were presented as percentages (%) for categorical variables and means [standard deviations (SDs)] or medians [interquartile ranges (IQRs)] for continuous variables. We used the chi-square test to compare differences in categorical variables and analysis of variance (ANOVA) to compare differences in continuous variables among the SARS-CoV-2 infection groups (i.e., confirmed COVID-19 diagnosis, probable COVID-19, and no COVID-19 infection). To calculate the cumulative incidence of SARS-CoV-2, we divided the number of SARS-CoV-2 cases by the number of study participants.

Univariable binary logistic regression analyses were performed to test the associations of SARS-CoV-2 infection with demographic variables, chronic medical conditions, and vaccination uptake status. All variables with *p* < 0.2 in the univariable logistic regression analyses were subsequently entered into a multivariable logistic regression model to determine the independent factors of SARS-CoV-2 infection. The results of the binary logistic regression analyses were presented as crude and adjusted odds ratios (ORs) along with their respective 95% confidence intervals (CIs). A significance level of *p* < 0.05 was used in the present study.

## Results

A total of 2,899 hospital staff members responded to the online questionnaire (response rate, 93.5%). Among the participants, 1,628 (56.2%) tested positive for SARS-CoV-2, of whom 1,331 (81.8%) used an antigen test, 398 (24.4%) used a PCR test, and 101 (6.2%) used both. Moreover, 877 (30.3%) staff members reported highly suspected COVID-19 symptoms, such as fever, dry cough and fatigue, but did not test for COVID-19. The cumulative incidence of SARS-CoV-2 infection between 7 December 2022 and 17 January 2023 was 86.4% (95% CI 85.2–87.7%), according to the case definition presented above.

Table 1 presents the demographic characteristics of the participants in the three groups (confirmed COVID-19 diagnosis, probable COVID-19, and no COVID-19 infection). The no COVID-19 infection group consisted of individuals who did not exhibit any COVID-19-like symptoms (*n* = 375, 95.2%), as well as those with suggestive COVID-19 symptoms but tested negative (*n* = 19, 4.8%). Among the 375 participants without any COVID-19-like symptoms, only 36 (9.6%) reported having a PCR or antigen test, all of which yielded negative results. The majority of patients received at least three doses of a COVID-19 vaccine (90.5%) and were without any chronic medical conditions (86.7%). Participants reporting no distinct symptoms tended to be male (*p* < 0.001), be 40–59 years old (*p* < 0.001), have a BMI of 24 kg/m^2^ or greater (*p* < 0.001), work in nonclinical areas (*p* < 0.001), have no need to work night shifts (*p* < 0.001) and have received at least three doses of vaccine with the most recent dose administered between one and six months prior to 7 December 2022 (*p* = 0.027), compared with individuals diagnosed with COVID-19 and those who were highly suspected of having COVID-19.

**Table 1:** Demographic and health characteristics of the participants

Variable	Total (*n* = 2899)		Confirmed COVID-19 diagnosis (*n* = 1628)		Probable COVID-19 (*n* = 877)		No COVID-19 infection (*n* = 394)		*p*- value^a^
	*n*	%	*n*	%	*n*	%	*n*	%	
**Gender**									
Male	567	19.6	280	17.2	154	17.6	133	33.8	< 0.001
Female	2332	80.4	1348	82.8	723	82.4	261	66.2	
**Age, years** ^**b**^									
Mean, SD	39.3	11.2	37.1	10.1	40.2	11.0	46.3	12.7	< 0.001
18–39	1646	57.0	1064	65.6	452	51.5	130	33.2	< 0.001
40–59	1126	38.9	523	32.2	397	45.3	206	52.6	
≥ 60	120	4.1	36	2.2	28	3.2	56	14.3	
**BMI, kg/m** ^**2 b**^									
Mean, SD	22.2	2.9	22.0	2.8	22.2	2.8	23.0	3.0	< 0.001
< 18.5	218	7.6	140	8.6	60	6.9	18	4.6	< 0.001
18.5–23.9	1944	67.3	1118	68.8	592	68.2	234	59.5	
≥ 24	725	25.1	368	22.6	216	24.9	141	35.9	
**Chronic medical conditions**									
No condition	2514	86.7	1418	87.1	744	84.8	352	89.3	0.168
One condition	342	11.8	188	11.5	119	13.6	35	8.9	
More than one condition	43	1.5	22	1.4	14	1.6	7	1.8	
**Occupation**									
Clinical practitioner	581	20.0	344	21.1	211	24.1	26	6.6	< 0.001
Nurse	1075	37.1	684	42.0	315	35.9	76	19.3	
Medical technician	272	9.4	180	11.1	72	8.2	20	5.1	
Administrative or logistics personnel	315	10.9	186	11.4	102	11.6	27	6.9	
Others (e.g. cleaning/food service, security, maintenance)	656	22.6	234	14.4	177	20.2	245	62.2	
**Working night shifts**									
No	1130	39.0	936	57.5	550	62.7	283	71.8	< 0.001
Yes	1769	61.0	692	42.5	327	37.3	111	28.2	
**COVID-19 vaccination Status**									0.027
< 3 doses	276	9.5	154	9.5	91	10.4	31	7.9	
≥ 3 doses and vaccinated in the past 1 month	112	3.9	64	3.9	30	3.4	18	4.6	
≥ 3 doses and vaccinated in the past 1–3 months	66	2.3	34	2.1	14	1.6	18	4.6	
≥ 3 doses and vaccinated in the past 3–6 months	129	4.4	70	4.3	35	4.0	24	6.1	
≥ 3 doses and vaccinated in the past > 6 months	2316	79.9	1306	80.2	707	80.6	303	76.9	

BMI: body mass index, SD: standard deviation

^a^ Chi-square test or ANOVA for differences among the three groups: confirmed COVID-19 diagnosis, probable COVID-19, and no COVID-19 infection

^**b**^ Missing data presented

**Fig. 1:**
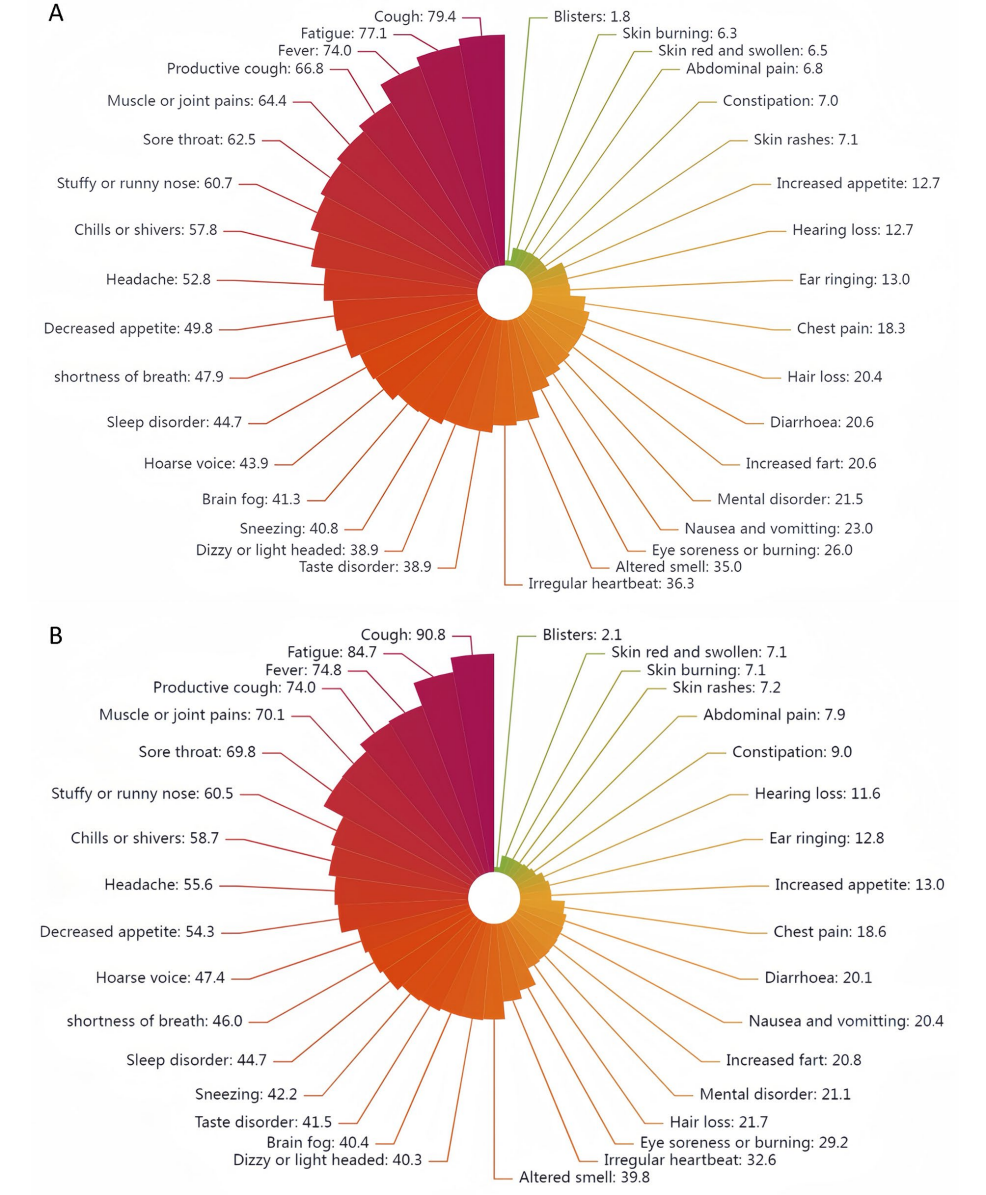
Symptoms reported by participants. **A**. Prevalence of symptoms reported by participants with confirmed COVID-19 diagnosis. **B**. Prevalence of symptoms reported by participants with highly suspected SARS-CoV-2 infection

Overall, cough (83.4%), fatigue (79.8%) and fever (74.3%) were the most prevalent symptoms. Among the 1,628 participants who tested positive, the most frequently reported symptoms were cough (1292 [79.4%]), fatigue (1255 [77.1%]), fever (1205 [74.0%]), productive cough (1088 [66.8%]), muscle or joint pain (1048 [64.4%]), sore throat (1018 [62.5%]) and stuffy or runny nose (988 [60.7%], Fig. 1a). Similarly, among the 877 participants without a diagnosis but who reported one or more potential symptoms of COVID-19, the most frequently reported symptoms were cough (796 [90.8%]), fatigue (743 [84.7%]), fever (656 [74.8%], productive cough (649 [74.0%]), muscle or joint pain (615 [70.1%]), sore throat (612 [69.8%]), and stuffy or runny nose (531 [60.5%], Fig. 1b).

Table 2 compares the COVID-19-related characteristics between individuals with a COVID-19 diagnosis and highly suspected COVID-19 patients. The former group reported a greater median number of COVID-19 symptoms (14, IQR [11–18]) and a higher percentage of individuals taking leave from work due to illness (44.5%) than did participants in the latter group (13, IQR [9–17]; 30%). However, no statistically significant differences in disease severity (using either admission to hospital or diagnosis of pneumonia as a proxy) were detected between the two groups (*p* > 0.05). Hospitalization was used as a surrogate measure of disease severity because it is typically reserved for patients with more severe and complex illnesses.

**Table 2:** COVID-19-related characteristics of the participants

Variable	Confirmed COVID-19 diagnosis (*n* = 1628)		Probable COVID-19 (*n* = 877)		*p* value^a^
	*n*	%	*n*	%	
**Number of symptoms, median (IQR)**	14	11–18	13	9–17	< 0.001
**Illness severity: admitted to hospital**					0.982
Not admitted to hospital	1613	99.1	869	99.1	
Admitted to hospital	15	0.9	8	0.9	
**Illness severity: diagnosis of pneumonia**					0.199
No	1469	90.2	805	91.8	
Yes	159	9.8	72	8.2	
**Took a leave from work due to the illness**					< 0.001
No	903	55.5	614	70.0	
Yes	725	44.5	263	30.0	

IQR: interquartile range

^a^Chi-square test or Mann‒Whitney U test for differences between two groups

The results of logistic regression analyses on COVID-19 infection are shown in Table 3. Based on the univariable analysis, the following six factors appeared to be potentially associated with SARS-CoV-2 infection: gender, age, BMI, occupation, working night shifts, and COVID-19 vaccination status. After adjusting for the effects of other confounding variables, the association between BMI and working night shifts was no longer significant. Compared with males, females were more likely (adjusted OR [aOR]: 1.42, 95% CI: 1.07–1.88) to be infected with SARS-CoV-2. Compared with those aged 18–39 years, the likelihood of SARS-CoV-2 infection decreased with age. Specifically, those aged ≥ 60 years had the lowest likelihood of infection (aOR: 0.30, 95% CI: 0.19–0.49). In terms of occupation, our study showed that clinical practitioners had the highest odds of SARS-CoV-2 infection (aOR: 10.32, 95% CI: 6.57–16.20) compared with those working in areas such as cleaning, security, and laundry. In addition, receiving at least three doses of the vaccine with the most recent dose administered within 3 months prior to 7 December 2022 decreased the likelihood of SARS-CoV-2 infection (aOR: 0.44, 95% CI: 0.23–0.87 for within 1 month; aOR: 0.46, 95% CI: 0.22–0.97 for within 1–3 months).

**Table 3:** Crude and adjusted odds ratios (95% confidence intervals) of SARS-CoV-2 infection among the participants

Variable	SARS-CoV-2 infection (*n* = 2505)		No SARS-CoV-2 infection (*n* = 394)		Crude OR (95% CI)	Adjusted OR (95% CI)^a^	*p* value^a^
**Gender**	*n*	%	*n*	%			0.015
Male	434	76.5	133	23.5	1.00	1.00	
Female	2071	88.8	261	11.2	2.43 (1.93, 3.07)	1.42 (1.07, 1.88)	
**Age, years** ^**b**^							< 0.001
18–39	1516	92.1	130	7.9	1.00	1.00	
40–59	920	81.7	206	18.3	0.38 (0.30, 0.48)	0.68 (0.51, 0.90)	
≥ 60	64	53.3	56	46.7	0.10 (0.07, 0.15)	0.30 (0.19, 0.49)	
**BMI, kg/m** ^**2 b**^							0.765
< 18.5	200	91.7	18	8.3	1.00	1.00	
18.5–23.9	1710	88.0	234	12.0	0.66 (0.40, 1.09)	0.96 (0.56, 1.63)	
≥ 24	584	80.6	141	19.4	0.37 (0.22, 0.63)	0.87 (0.49, 1.54)	
**Chronic medical conditions**							0.090
No condition	2162	86.0	352	14.0	1.00	1.00	
One condition	307	89.8	35	10.2	1.43 (0.99, 2.06)	1.50 (1.00, 2.26)	
More than one condition	36	83.7	7	16.3	0.84 (0.37, 1.90)	0.68 (0.28, 1.64)	
**Occupation**							< 0.001
Clinical practitioner	555	95.5	26	4.5	12.73 (8.33, 19.44)	10.32 (6.57, 16.20)	
Nurse	999	92.9	76	7.1	7.84 (5.91, 10.39)	5.40 (3.81, 7.67)	
Medical technician	252	91.4	20	8.6	6.36 (4.16, 9.73)	5.98 (3.83, 9.32)	
Administrative or logistics personnel	288	92.6	27	7.4	7.51 (4.64, 12.16)	5.64 (3.39, 9.36)	
Others (e.g. cleaning/food service, security)	411	62.7	245	37.3	1.00	1.00	
**Working night shifts**							0.449
No	1486	84.0	283	16.0	1.00	1.00	
Yes	1019	90.2	111	9.8	1.75 (1.39, 2.21)	1.11 (0.84, 1.47)	
**COVID-19 vaccination Status**							0.001
< 3 doses	245	88.8	31	11.2	1.00	1.00	
≥ 3 doses and vaccinated in the past 1 month	94	83.9	18	16.1	0.66 (0.35, 1.24)	0.44 (0.23, 0.87)	
≥ 3 doses and vaccinated in the past 1–3 months	48	72.7	18	27.3	0.34 (0.18, 0.65)	0.46 (0.22, 0.97)	
≥ 3 doses and vaccinated in the past 3–6 months	105	81.4	24	18.6	0.55 (0.31, 0.99)	1.05 (0.55, 2.02)	
≥ 3 doses and vaccinated in the past > 6 months	2013	86.9	303	13.1	0.84 (0.57, 1.25)	1.19 (0.77, 1.84)	

BMI: body mass index, OR: odds ratio, 95% CI: 95% confidence interval

^a^ Adjusted for other variables in the table

^b^ Missing data presented

All 2,505 SARS-CoV-2-infected cases were successfully followed up with telephone interviews. Among them, 170 (4.27%) reported experiencing either fatigue, brain fog or both for at least two months after the initial illness. Among the 80 participants with long COVID symptoms of fatigue, a small proportion reported severe symptoms. More specifically, 13.8%, 11.3% and 16.3% of respondents rated the symptoms as severe for the three fatigue items “feeling of physical or mental exhaustion that does not improve with rest”, “being interested and wanting to do things but not having the energy” and “worsening of fatigue following simple physical or mental activities”, respectively. Additionally, among the 100 participants reporting at least one symptom of brain fog, only a few considered their condition moderate/severe (or sometimes/always) in terms of forgetfulness (31.0%), poor concentration (24.0%), fuzzy thoughts (4.0%), lost words (2.0%), feeling sluggish (1.0%), and feeling confused (none). Of the 2,505 infected participants, 23 (0.92%) experienced at least one severe symptom of long COVID.

## Discussion

Our study revealed a cumulative incidence of SARS-CoV-2 Omicron infection among hospital staff from Chengdu Women’s and Children’s Hospital to be 86.4% (95% CI 85.2%-87.7%), which was higher than the reported rate of 74.3% among the general population in the same province [18]. However, since our study was conducted more than one week later, it is uncertain whether hospital staff faced a greater infection risk compared to individuals in the community or if the timing difference influenced the documentation of cumulative incidence rates during a rapid and widespread Omicron outbreak. In addition, our rate was much higher than that previously reported for healthcare workers in other studies, most of which had an even longer time frame than our research [19–23]. According to a study conducted in Japan, the cumulative incidence of infection among healthcare workers increased substantially from 2.0% in June 2021 (pre-Delta) to 39.0% in December 2022 (Omicron variant-predominant period) [19]. Similarly, the infection rate of healthcare workers in China remained low before the emergence of the Omicron variants [24]. No previous studies have reported the epidemiology of COVID-19 infection among healthcare workers during the Omicron outbreak in mainland China. A study from Hong Kong revealed that nearly one in five healthcare workers were infected between December 2021 and May 2022, when the Omicron sublineages caused outbreaks in the community [8]. The extremely high infection rate of hospital staff observed in our study was in line with the surge of COVID-19 cases in China around the end of 2022, when the ‘dynamic zero-COVID policy’ was eased and Omicron subvariant BA.5 was dominant [25].

We observed a high prevalence of systemic symptoms, including fatigue and fever, among our participants who had either a confirmed COVID-19 diagnosis or highly suspected symptoms. These clinical manifestations were different from those reported in other studies [15, 26]. For instance, a registry-based observational study in Japan revealed that when the omicron subvariants BA.2 and BA.5 were prevalent, upper respiratory symptoms such as cough (62.7%), sore throat (60.7%) and nasal discharge (44.3%) were more common than fever (38.8%) and severe fatigue (26.8%) [26]. A large proportion of the disparities in clinical presentation may be attributed to differences in previous infection status. A history of previous SARS-CoV-2 infection has been reported to be inversely associated with the risk of systemic symptoms [26, 27]. During the implementation of the ‘dynamic zero-COVID strategy’, China witnessed an exceptionally low infection rate. with no cases detected among our hospital staff. A lack of innate immune protection following SARS-CoV-2 infection, irrespective of vaccine-induced immunity, potentially contributes to the heightened prevalence of fatigue and fever in our study participants. In addition, the high prevalence of systemic symptoms in the present study aligns with the findings of a previous survey conducted on 328 healthcare workers infected with the Omicron variants, in which 83.5% reported experiencing fever [28]. The self-reporting of healthcare workers, who possess sufficient knowledge and awareness to monitor their own body temperature, may have played a significant role. Furthermore, considering the escalated disease burden of COVID-19 in China, including within work environments, it is plausible that the participants were highly conscious of potential symptoms, which could have influenced their reporting.

In our study, female staff members had a greater risk of SARS-CoV-2 infection than did their male colleagues. This finding adds to the evidence of gender disparities in COVID-19 susceptibility among the prime working-age population. Accumulating epidemiological evidence has shown similar infection rates of SARS-CoV-2 between males and females in general communities [29]. Nevertheless, a study using data reported by health authorities in Canada revealed that working-age women were more vulnerable to infections than were similar-aged men during the COVID-19 pandemic [30]. Women’s predominant roles as caregivers both in families and workforces may expose them to an elevated risk of infection [31].

Consistent with some previous research [20, 32], we found that older individuals were less likely to become infected. It has been widely recognized that older COVID-19 patients, especially those aged over 65 years, have strikingly greater mortality rates than younger individuals [33, 34]. Thus, to reduce possible adverse consequences, older hospital staff may have higher compliance with the implementation of preventive measures.

Being a clinical practitioner greatly increased the risk of contracting COVID-19 compared with working as a cleaner, secure guard or maintenance worker in hospitals, who have little direct contact with patients or medical professionals. During the emerging outbreaks at the end of 2022, hospitals in Chengdu were overwhelmed with patients seeking medical care. Clinical practitioners inevitably have a large number of contacts with COVID-19 patients, leading to high levels of stress and workloads. These staff may then reduce their compliance with protective measures and consequently a high rate of SARS-CoV-2 infection. Moreover, no significant differences in infection rates were found among clinical practitioners, nurses, medical technicians and administrative/logistic personnel. The intensive cooperation of relevant departments within the hospital might contribute to explaining these findings. Since there was frequent interdepartmental support during the surge of COVID-19 patients, we did not further divided the clinical practitioners and nurses into specific departments.

COVID-19 vaccination is a safe and effective way to prevent related hospitalizations and deaths. By May 2022, three Chinese COVID-19 vaccines, two inactivated and one recombinant, had been listed by the WHO for emergency use [35]. Nine out of ten staff members in our hospital had received at least three doses of COVID-19 vaccines, but 88.3% of them had the latest dose administered more than 6 months before the change in the COVID-19 containment policy. A study from Hong Kong showed a significant decrease in neutralizing antibodies 4 months after vaccination in a group of people who received the inactivated CoronaVac vaccine [35]. Therefore, it was not surprising to find that a three-dose vaccination, with the most recent dose administered within 3 months before 7 December 2022, was associated with a reduced risk of SARS-CoV-2 infection in our study.

Among the SARS-CoV-2 infection patients, all of whom were infected for the first time, the incidence of long COVID-related fatigue or brain fog was 4.27%. It was comparatively lower than that observed in the majority of other studies on long COVID among both the general population and healthcare workers [13, 36]. The disparities were likely attributed to our inclusion of only two symptoms. For instance, the corresponding rate was 8.89% in a study conducted during the Omicron BA.2 outbreak in Shanghai, China [37]. However, it is important to note that the Shanghai study encompassed 18 pre-specified long COVID symptoms. In another study of 679 healthcare workers who tested positive for COVID-19, nearly one-third reported having suffered from at least one of the 40 long COVID symptoms assessed [38]. In addition, previous studies have shown that no vaccination, severe COVID-19 illness during the acute phase, advanced age and obesity are common factors associated with an increased risk of long COVID [36, 39]. Thus, the relatively low incidence of long COVID symptoms in our study may also be partially attributed to the high prevalence of three-dose vaccination (90.2%), low incidence of hospitalization (0.9%), and low prevalence of advanced age (≥ 60 years) and obesity (≥ 28 kg/m^2^) (both < 4%) among our SARS-CoV-2 infection patients.

Our study has several limitations. First, data on confirmed SARS-CoV-2 infection were unavailable since large-scale nucleic acid testing was overhauled and there was a national shortage of COVID-19 antigen test kits at the end of 2022. The use of self-reported data may introduce some misclassification. However, since there were no flu or other respiratory disease (e.g., respiratory syncytial virus) waves in Chengdu throughout the last winter, the number of misclassified cases should be extremely small. Second, long COVID includes a broad range of symptoms; however, due to logistic reasons, we only focused on fatigue and brain fog. This may lead to an underestimation of the risk of long COVID. In addition, symptom data are subject to variance in individual perceptions. Some participants may be more prone to report the presence of symptoms than others. Nevertheless, it was unlikely that significant recall errors occurred in our study, as the baseline information was collected within a short period of the outbreak, with follow-up interviews conducted between five and six months after the initial infection. The telephone follow-up interviews were conducted by trained research assistants to ensure a standardized data collection process and minimize potential bias. Furthermore, a recent meta-analysis highlighted the occupational exposures associated with COVID-19 infection among healthcare workers, such as inadequate or lack of personal protective equipment and performing tracheal intubation [10]. However, the present study did not gather data on these specific factors, potentially leading to unmeasured confounding that cannot be adjusted for in the regression analyses. Finally, since we recruited participants from a single hospital, our sample may not be representative of the target population in China.

## Conclusion

Our findings offer a valuable snapshot of the epidemiological situation regarding first-time SARS-CoV-2 Omicron infection among healthcare workers in mainland China, particularly following China’s deregulation of COVID-19 control measures. The current study can help to understand how hospital staff were impacted by the virus in the context of evolving control strategies. Our data have potential implications for informing targeted interventions and preventive measures within healthcare systems, particularly during a rapid and widespread Omicron outbreak.

## Data Availability

Please contact the corresponding author for data requests.

## Abbreviations

ANOVA: Analysis of variance
BMI: Body mass index
CIs: Confidence intervals
COVID-19: Coronavirus Disease 2019
ORs: Odds ratios
PCR: Polymerase chain reaction
SARS-CoV-2: Severe Acute Respiratory Syndrome coronavirus-2
SD: Standard deviation
